# Comparison of different input modalities and network structures for deep learning-based seizure detection

**DOI:** 10.1038/s41598-019-56958-y

**Published:** 2020-01-10

**Authors:** Kyung-Ok Cho, Hyun-Jong Jang

**Affiliations:** 1 Department of Pharmacology, Department of Biomedicine & Health Sciences, Catholic Neuroscience Institute, College of Medicine, The Catholic University of Korea, Seoul, 06591 South Korea; 2 Department of Physiology, Department of Biomedicine & Health Sciences, Catholic Neuroscience Institute, College of Medicine, The Catholic University of Korea, Seoul, 06591 South Korea

**Keywords:** Data processing, Diagnostic markers

## Abstract

The manual review of an electroencephalogram (EEG) for seizure detection is a laborious and error-prone process. Thus, automated seizure detection based on machine learning has been studied for decades. Recently, deep learning has been adopted in order to avoid manual feature extraction and selection. In the present study, we systematically compared the performance of different combinations of input modalities and network structures on a fixed window size and dataset to ascertain an optimal combination of input modalities and network structures. The raw time-series EEG, periodogram of the EEG, 2D images of short-time Fourier transform results, and 2D images of raw EEG waveforms were obtained from 5-s segments of intracranial EEGs recorded from a mouse model of epilepsy. A fully connected neural network (FCNN), recurrent neural network (RNN), and convolutional neural network (CNN) were implemented to classify the various inputs. The classification results for the test dataset showed that CNN performed better than FCNN and RNN, with the area under the curve (AUC) for the receiver operating characteristics curves ranging from 0.983 to 0.984, from 0.985 to 0.989, and from 0.989 to 0.993 for FCNN, RNN, and CNN, respectively. As for input modalities, 2D images of raw EEG waveforms yielded the best result with an AUC of 0.993. Thus, CNN can be the most suitable network structure for automated seizure detection when applied to the images of raw EEG waveforms, since CNN can effectively learn a general spatially-invariant representation of seizure patterns in 2D representations of raw EEG.

## Introduction

Epilepsy is defined as having unprovoked recurrent seizures^1,2^. The primary tool for seizure detection is the electroencephalogram (EEG). EEG continuously measures the electrical activity of the brain via electrodes placed on the scalp or the surface of the brain. Manual inspection of long, continuous EEGs for seizure detection is a time consuming and laborious process in both clinical and experimental settings. It can take many hours to meticulously examine days of EEG recordings for patients hospitalized to diagnose epilepsy. In an experimental setting, long-term EEG recordings (even up to several months) are often to be reviewed. Furthermore, the EEG readings made by different inspectors can be inconsistent as the criteria for abnormal EEG findings are experiential^3^. Therefore, the development of an automated method for seizure detection is necessary.

For decades, various machine learning approaches have been applied to detect seizures automatically^4,5^. The difficulty in automatic seizure detection is due to the extreme variability in both inter- and intra-patient EEG^6^. Furthermore, EEG signals are highly non-stationary and nonlinear^7,8^. Thus, to construct a generalized seizure detector, discriminative features between seizure and non-seizure EEGs should be extracted. Many existing methods have been based on hand-engineered techniques for extracting features in the time domain, frequency domain, time-frequency domain, and using combinations of multiple domains from EEG signals^5^. Time domain features include average wave amplitude and duration, coefficient of variation in the wave amplitude and duration, and skewness and kurtosis^9^. Frequency domain features can be obtained by a fast Fourier transform (FFT) or periodogram^10–12^. Time-frequency domain features can be extracted by a short-time Fourier transform (STFT) or wavelet transform^13–16^. Nonlinear analysis, including an entropy-based approach, has also been used to extract features^17,18^. Many studies adopted combinations of multiple domain features to enhance their classification results^19,20^. These extracted features were then statistically analyzed, ranked, and classified. The best classifier was determined by comparing the performance of different classifiers for selected features. The types of classifiers have included an artificial neural network^21^, k-nearest neighbor^16^, logistic regression^20^, naïve Bayes^13^, random forest^3^, and support vector machine^10^. Thus, machine learning-based seizure autodetection was traditionally composed of two separate procedures. The first part was the feature extraction process and the other was the classification process applied to the extracted features. Both the identification of the appropriate features and the choice of a proper classifier can play important roles in optimizing algorithm performance. These processes depend heavily on domain expertise and consume a great deal of time and effort to select proper features and classifiers.

Thus, automatic feature learning has substantial advantages over traditional machine learning methods based on manual feature extraction and selection^22^. This can be accomplished by the implementation of deep learning, which automatically discovers and learns the discriminative features needed for the classification of inputs^23^. Recently, many studies have investigated deep learning for seizure detection. These studies have been based on different deep neural network structures, such as a fully connected neural network (FCNN)^24^, convolutional neural network (CNN)^22,25–27^, and recurrent neural network (RNN)^28^. These different neural networks can automatically learn discriminative features from various types of data input, including raw temporal EEG^26^, FFT results^25^, 2-dimensional (2D) representation of STFT results^29^, and 2D images of raw EEG^27^. The adoption of different input forms and network structures typically makes it difficult to directly compare performance among different deep learning methods. Furthermore, these studies adopted different window sizes for EEG segmentation (e.g., 1-^25^, 2-^29^, 3-^22^, 5-^24,27^, 8-^26^, and 23.6-s^28^ windows). In addition, the classifiers were trained and tested on different datasets including public EEG datasets such as the Bonn^22,28^, CHB-MIT^25,29^, and Freiburg^25^ datasets, or their own datasets^24,27^. Thus, it is almost impossible to directly compare the results of different studies to ascertain an optimal combination of input modalities and network structures.

Therefore, in the present study, we compared the performance of deep learning-based seizure detection algorithms using combinations of different input forms and network structures to systematically investigate how the input modalities and network structures can affect the characteristics of automated seizure detectors. Since previous studies adopted the time, frequency, and time-frequency domain signals and the images of EEG as inputs, we decided to include all these input domains to meticulously search for the most suitable input forms. Thus, the raw time-series and periodogram of EEGs, 2D images of STFT results, and 2D raw EEG waveform images, which were obtained from experimental intracranial EEG (iEEG) traces in a mouse model of epilepsy, were adopted as input forms. Every input was made from a 5-s segment EEG trace. Since the FCNN, RNN, and CNN have been widely used for EEG classification, we included these network structures to classify our input data. For the raw time-series and periodogram of EEGs, all three networks were applied. For the 2D image inputs, only the CNN was applied. Thus, nine possible combinations of input modalities and network structures were tested in the present study. We considered the nine combinations can provide decent comparison for the classification performance of the widely adopted input modalities and network structures. Then, we tested three previously reported classifiers on our experimental iEEG to compare with the results of the current study. Finally, our classifiers were tested on a human iEEG dataset to validate the results of this study.

## Methods

### EEG recording

The iEEGs used in the present study were recorded from mice for epilepsy research. The animal experiments were approved by the Ethics Committee of the Catholic University of Korea and were carried out in accordance with the National Institutes of Health Guide for the Care and Use of Laboratory Animals (NIH Publications No. 80-23). Details of establishing the mouse model of pilocarpine-induced epilepsy were the same as previously described^30,31^. Between 4 and 7 weeks after pilocarpine injection, video/EEG monitoring was conducted for 2 weeks as previously described^32,33^. Each mouse was stereotactically implanted with a single epidural recording electrode, placed at AP −0.2 mm and ML +0.22 mm from bregma. Reference electrode was implanted at AP +0.1 mm and ML +0.1 mm from bregma. Mice underwent continuous monitoring by a wireless video/EEG monitoring system (Data Sciences International). An expert epileptologist evaluated all the EEG traces to detect generalized tonic-clonic seizures with the baseline suppression and the restoration of the EEG amplitudes to the baseline as the onset and offset of the seizures, respectively. Convulsive seizures were further defined by repetitive epileptiform spiking (≥3 Hz) that persisted for more than 3 s and were also confirmed by video recordings.

### Datasets

**Figure 1:**
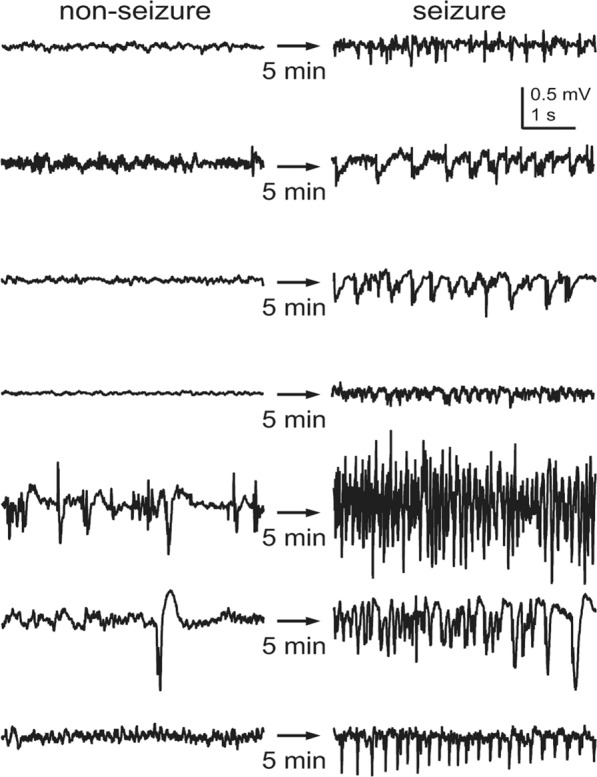
Non-seizure and seizure segments obtained from seven different recordings. Both showed extremely variable patterns. The non-seizure segment was recorded 5 min before each seizure segment.

We obtained the training and test sets from completely separate groups of mice. Single channel iEEG data recorded from 17 mice (total 4,704 h) were used as the training set, which contained 249 human-annotated seizure events ranging from 8.34 to 61.25 s in duration^24^. The test set consisted of 4,272 h of EEG recordings from another 15 mice, containing 324 seizure events. To construct a training dataset, we collected seizure EEG segments from annotated seizure events using a 5-s sliding window with a 0.25-s interval. Non-seizure segments were collected from 5 min of EEG traces before and after each seizure event using a 5-s sliding window with a 2.5-s interval. We used different intervals for the collection of seizure and non-seizure segments because seizure EEGs were relatively scarce. The total numbers of training segments were 15,828 and 46,753 for the seizure and non-seizure, respectively. We chose to analyze the 5-s segment of EEGs based on our previous study because it was the most efficient for seizure event detection^24^. Both seizure and non-seizure segments showed extremely varied patterns (Fig. 1), suggesting that it is a very challenging task to extract general features.

### Input forms

**Figure 2:**
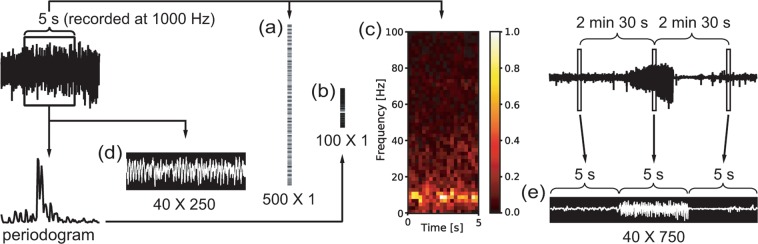
The different input forms used in the present study. (**a**) A down-sampled raw time-series EEG with 500 data points. (**b**) Periodogram result with 100 data points. (**c**) Image of an STFT at 50 × 20 pixels. The gray STFT image was pseudo-colored for demonstration purposes. (**d**) Image of an EEG waveform at 40 × 250 pixels. (**e**) Concatenated image of three temporally separated images of EEG waveforms at 40 × 750 pixels. The images are not presented to reflect their actual sizes.

The 5-s segments were transformed into different forms for the input into deep neural networks (Fig. 2). The original iEEG was recorded at 1,000 Hz. It was down-sampled to 100 Hz by averaging sampling, thus resulting in 500 raw EEG data points for 5-s segments (Fig. 2a). A periodogram between 0 and 99 Hz (100 data points) was analyzed from the 5-s segments of the original EEGs (Fig. 2b). The 5-s segments were also transformed into a gray image by an STFT using the Hamming window (Fig. 2c). Finally, the raw EEG waveform was transformed into a black and white image with dimensions of 40 × 250 pixels (Fig. 2d). In order to more accurately capture the characteristics of seizure EEGs that markedly differ from pre- and post-convulsive EEG patterns^4^, we additionally constructed the last input form by concatenating three black and white images of 5-s EEG segments, which were separated by a 2.5-min interval (Fig. 2e). Thus, the last input form became a set of 40 × 750 pixel-images of the EEG. Because the longest seizure was approximately 60 s in our experimental data set, a 2.5-min interval could clearly separate seizure and non-seizure EEGs. In summary, a total of five different input forms, i.e., a down-sampled raw EEG time-series with 500 data points, periodogram results with 100 data points, 50 × 20 pixel gray images of STFT, and black and white images of raw EEG with dimensions of either 40 × 250 pixels or 40 × 750 pixels, were used for the deep learning-based seizure autodetectors.

### Network structures

**Figure 3:**
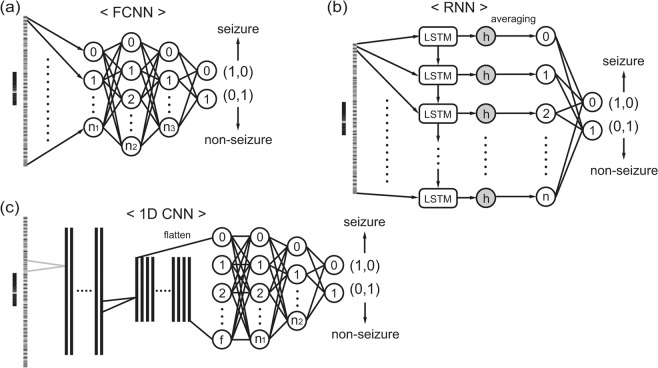
Network structures used to classify a down-sampled raw time-series EEG and periodogram result. (**a**) A fully connected neural network (FCNN). **(b**) Recurrent neural network (RNN) implemented with a long short-term memory (LSTM) cell. (**c**) Convolutional neural network (CNN) for 1-dimensional (1D) input.

**Figure 4:**
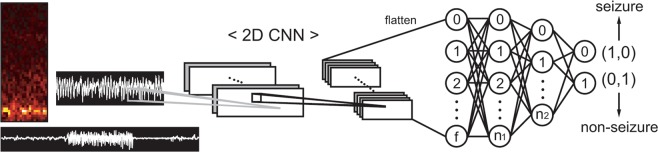
The network structure for 2-dimensional (2D) images. A convolutional neural network (CNN) was used for 2D input.

Three different network structures, including the FCNN, RNN, and 1D CNN were used to construct seizure detectors from the raw EEG time-series and periodogram results (Fig. 3). We adopted a simple grid search strategy to determine the most suitable network structures during training. We generally tested only three to four values for each parameter because there were too many combinations of input modalities and network structures to perform an extensive search. Two to four layers with various node numbers were tested for the FCNN. For the RNN, unit size for the memory cell was searched. For the CNN, kernel size and the number of convolution layers were grid-searched but the strides were fixed as 1 and 2 for the convolution and pooling layers, respectively. We adopted the simplest structures that yielded the best results. Furthermore, three minibatch sizes were tested for each structure: 64, 128, and 256 samples per minibatch. The FCNN consisted of two hidden layers and an output layer containing two nodes for seizure and non-seizure classification (Fig. 3a). Because the current problem is binary classification, only one node can be used for the output layer. However, we assigned nodes per class for the future implementation of the multi-class problem (i.e., interictal, preictal, and ictal classification). The RNN was implemented with a long short-term memory (LSTM) cell (Fig. 3b). We averaged the outputs from the LSTM cell for each input sequence to construct a layer before the output layer, similar to the structure used in the work by Hussein *et al*. Every two consecutive data points in the input data were passed to the LSTM cell. Thus, the down-sampled raw EEG and periodogram yielded 250 and 50 sequence lengths for the RNN, respectively. The LSTM unit size was determined to be 20. Since the raw time-series EEG and periodogram results were basically 1D data forms, we implemented 1D CNNs for these inputs (Fig. 3c). For both input forms, two consecutive convolutional-pooling layers were sufficient to yield the best results. The outputs of the second pooling layer were flattened and passed to two fully-connected layers. For the gray images of the STFT and black and white images of the raw EEG waveform, only 2D CNNs were applied to construct seizure detectors (Fig. 4). As in the case of the 1D CNN, two consecutive convolutional-pooling layers were adequate and achieved the best results. The details of the network structures are summarized in Supplementary Table 1. The deep neural networks were implemented with the TensorFlow deep learning library (https://www.tensorflow.org). A dropout was applied on every fully connected hidden layer in each network structure with a ratio of 0.3. The networks were trained on minibatches with sizes ranging from 128 to 256 using the Adam optimizer with default parameters (learning rate: 0.001, decay rate of the first and the second moments: 0.9 and 0.999). Ten percent of training data was randomly selected as a validation set and the validation loss was used as an early stopping criterion to avoid overfitting.

### Seizure event detection

In our previous study, we built a seizure event detector based on the classifier for the 5-s EEG segments^24^. In the present study, we adopted the same procedure to detect seizure events. Briefly, the 5-s EEG segments were continuously classified and nearby seizure segments were joined to form a seizure event when separated seizure segments were detected within 10 s in continuous EEGs. Single discrete seizure segment was removed. Then, if the seizure event had 1.2 times higher mean absolute amplitude compared to nearby EEG, the event was finalized as a seizure event. If there was no amplitude change, the event was not considered as a seizure event. This post-processing effectively eliminated much of false positive (FP) segments. The mean absolute amplitude was used to meaningfully estimate EEG amplitudes because positive and negative signals in the EEG can negate each other if they are not converted to absolute values. When the detected event did not overlap with human annotated seizure events, it was considered as a false detection.

### Validation of models with human iEEGs

To validate the performance difference in the present study, we tested our models on a human iEEG dataset which was used for ‘UPenn and Mayo Clinic’s Seizure Detection Challenge’ held at kaggle.com (https://www.kaggle.com/c/seizure-detection). There were iEEGs recorded from 8 patients with channel numbers ranging from 16 to 72. Sampling frequency was 5,000 Hz except for 500 Hz in patient 1. The dataset offered the EEGs as the independent files of 1-s EEG segments. The 1-s segments were divided into ictal, interictal, and test sets. Because we did not have the answer for the test set, we only used the ictal and interictal sets in the current study. We set the frequency to 500 Hz and concatenated five of the continuous 1-s segments to build a 5-s segment of EEG. Because the minimum channel was 16, we selected 16 channels to train the seizure classifiers for every patient. We built classifiers for the periodogram with 1D CNN, for the images of the STFT with 2D CNN, and for the images of raw EEG waveform with 2D CNN. We tested only the CNN-based classifiers because the multi-channel data can be easily incorporated with the data channels in CNNs. Both intra- and inter-patient classification were tested. Intra-patient classification was performed with 10-fold cross-validation scheme for each patient. Because there were only 8 patients, inter-patient classification was 8-fold cross-validated, i.e., classifiers trained with 7 patients were tested on the remaining one patient. To draw the ROC curves, the results for all folds were concatenated.

### Performance metrics and statistics

The true positive (TP) denotes the seizure segment when it is classified as a seizure by a deep neural network. The false negative (FN) is the seizure segment falsely classified as a non-seizure. The true negative (TN) and FP are non-seizure segments classified as a non-seizure and falsely as a seizure, respectively. TP, FN, TN, and FP numbers were presented as mean ± SE. One-way analysis of variance (ANOVA) and Tukey’s post hoc test were used to compare the numbers. The accuracy, sensitivity, specificity, and F1 score are well-known performance metrics used to evaluate the performance of a binary classifier, and are calculated as follows:

$$\begin{array}{rcl}sensitivity & = & \frac{{\rm{TP}}}{{\rm{TP}}+{\rm{FN}}}\\ specificity & = & \frac{{\rm{TN}}}{{\rm{FP}}+{\rm{TN}}}\\ accuracy & = & \frac{{\rm{TP}}+{\rm{TN}}}{{\rm{TP}}+{\rm{FN}}+{\rm{FP}}+{\rm{TN}}}\\ F1\,score & = & \frac{2\cdot {\rm{TP}}}{2\cdot {\rm{TP}}+{\rm{FP}}+{\rm{FN}}}\end{array}$$

A receiver operating characteristics (ROC) curve was plotted for every classifier and the difference between the two ROC curves was evaluated by the permutation test using 1,000 permutations^34^. To compare the performance for event detection, false detection rate (FDR) per hour (number of falsely detected seizure events/hour) was also presented. A *p*-value <0.05 was considered significant.

## Results

We trained each combination of input forms and network structures five times and let them classify the 5-s EEG segments collected at 2.5-s intervals from 4,272 h of the test data set (n = 5 for every classifier). The total number of test segments for the seizures and non-seizures were 928 and 3,804,948, respectively. There were slightly fewer segments for concatenated raw EEG images with dimensions of 40 × 750 pixels (seizure: 923 and non-seizure 3,730,236).

**Figure 5:**
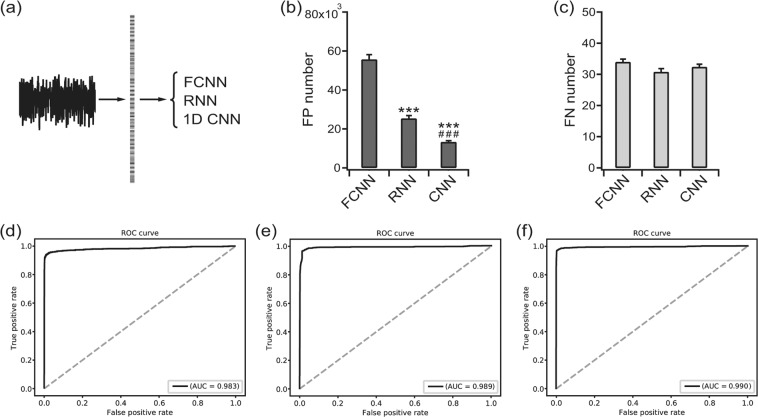
Classification results for the down-sampled raw time-series EEGs. (**a**) The inputs were classified with a fully connected neural network (FCNN), recurrent neural network (RNN) and convolutional neural network (CNN) for 1D input. (**b**) False positive (FP) numbers for the FCNN, RNN, and CNN. ***p  < 0.001 vs. FCNN, ###*p*  < 0.001 vs. RNN. (**c**) False negative (FN) numbers for the FCNN, RNN, and CNN. (**d**) The receiver operating characteristics (ROC) curve for the classification result of the FCNN. (**e**) The ROC curve for the classification result of the RNN. (**f**) The ROC curve for the classification result of the 1D CNN. The area under the curve (AUC) is presented for each ROC curve.

**Figure 6:**
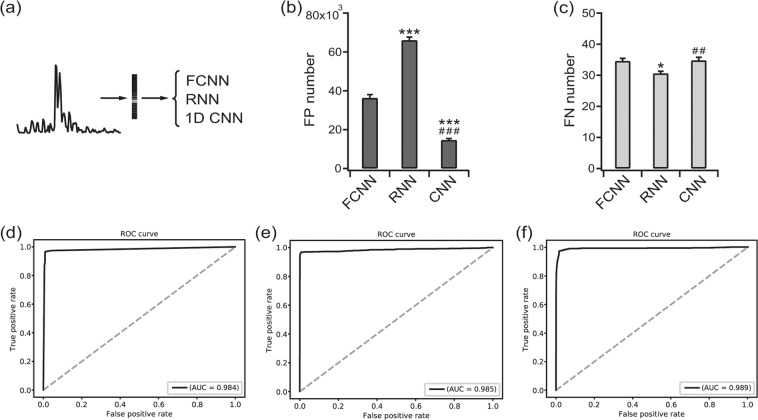
Classification results for the periodogram. (**a**) The inputs were classified using a fully connected neural network (FCNN), recurrent neural network (RNN) and convolutional neural network (CNN) for 1D input. (**b**) False positive (FP) numbers for the FCNN, RNN, and CNN. ***p  < 0.001 vs. FCNN, ^###^*p*  < 0.001 vs. RNN. (**c**) False negative (FN) numbers for the FCNN, RNN, and CNN. *p  < 0.05 vs. FCNN, ^##^*p*  < 0.01 vs. RNN. (**d**) The receiver operating characteristics (ROC) curve for the classification result of the FCNN. (**e**) The ROC curve for the classification result of the RNN. (**f**) The ROC curve for the classification result of the 1D CNN. The area under the curve (AUC) is presented for each ROC curve.

We first applied the FCNN, RNN, and 1D CNN to raw time-series EEGs (Fig. 5a). The FP numbers decreased in the order of the FCNN, RNN, and 1D CNN as 55,695.8 ± 2,377.05, 25,349.8 ± 1,464.69, and 13,314.8 ± 610.87 [F(2, 12) = 175.2, *p* < 0.001, one-way ANOVA], respectively (Fig. 5b). The FN numbers among the network structures were not significantly different: 33.6 ± 0.67, 30.8 ± 0.91, and 32.4 ± 0.81 for the FCNN, RNN, and 1D CNN [F(2, 12) = 2.741, *p* = 0.104, one-way ANOVA], respectively (Fig. 5c). The ROC curve yielded an AUC of 0.983 (Fig. 5d), 0.989 (Fig. 5e), and 0.990 (Fig. 5f) for the FCNN, RNN, and 1D CNN, respectively (*p* = 0.039 for the FCNN vs. RNN, *p* = 0.012 for the FCNN vs. 1D CNN and *p* = 0.327 for the RNN vs. 1D CNN by the permutation test). When the FCNN, RNN, and 1D CNN were applied to periodogram results (Fig. 6a), the pattern was more complex. The FP numbers fluctuated with the FCNN, RNN, and 1D CNN yielding 36,345.0 ± 1,706.69, 66,044.4 ± 1,594.71, and 14,669.2 ± 757.95 [F(2, 12) = 330.9, *p* < 0.001, one-way ANOVA], respectively (Fig. 6b). The FN number showed a reversed pattern with 34.6 ± 0.81, 30.6 ± 0.67, and 34.8 ± 0.96 for the FCNN, RNN, and 1D CNN [F(2, 12) = 8.175, *p* < 0.01, one-way ANOVA], respectively (Fig. 6c). The AUCs were 0.984 (Fig. 6d), 0.985 (Fig. 6e), and 0.989 (Fig. 6f) for the FCNN, RNN, and 1D CNN, respectively (*p* = 0.382 for the FCNN vs. RNN, *p* = 0.052 for the FCNN vs. 1D CNN, and *p* = 0.117 for the RNN vs. 1D CNN by the permutation test).

**Figure 7:**
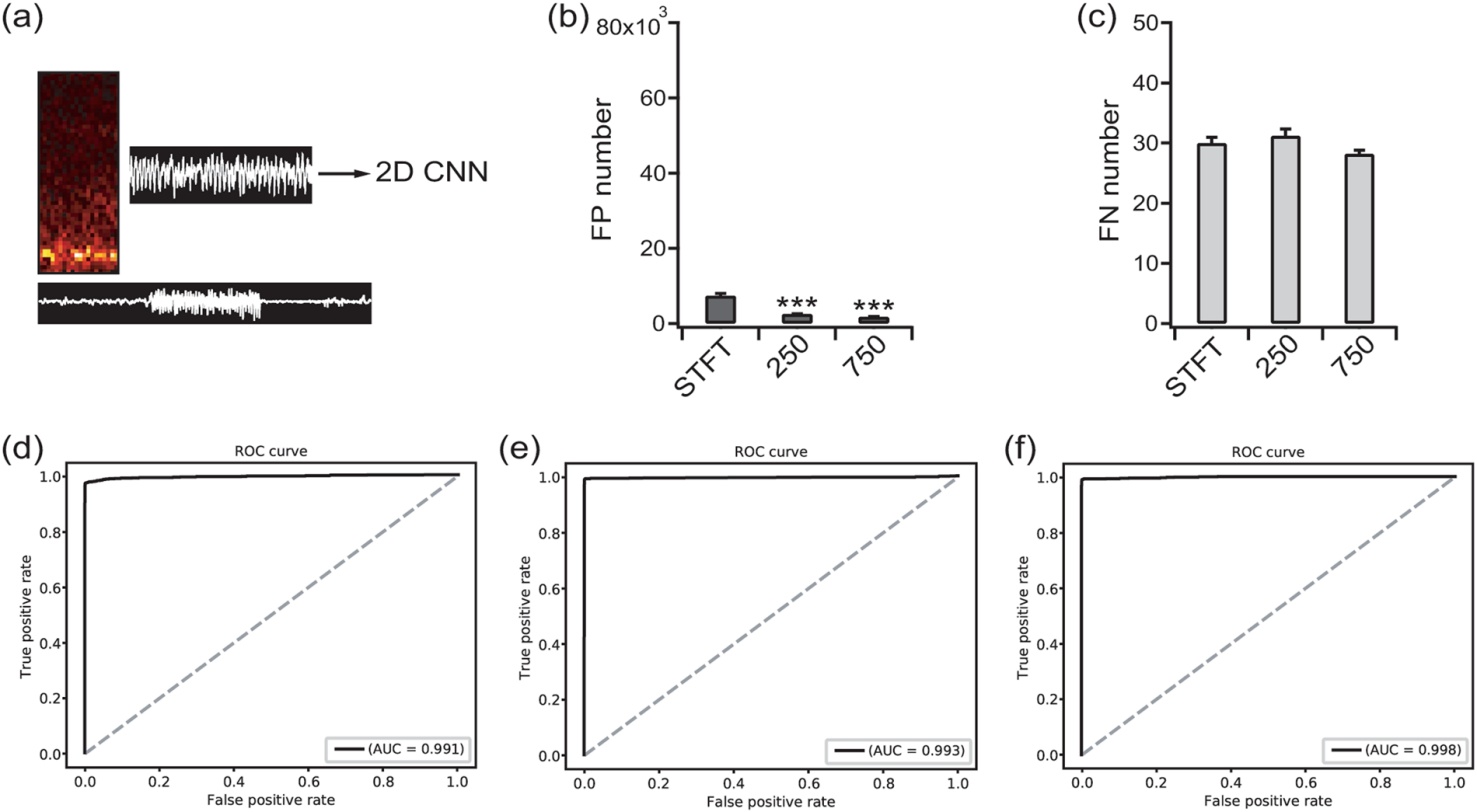
Classification results for the STFT images at 50 × 20 pixels, images of the EEG waveform at 40 × 250 pixels and the concatenated images of three temporally separated images of EEG waveforms at 40 × 750 pixels. (**a**) The inputs were classified with a convolutional neural network (CNN) for 2D input. (**b**) The false positive (FP) numbers for the STFT images, waveform images at 40 × 250 pixels and waveform images at 40 × 750 pixels. ***p  < 0.001 vs. STFT. (**c**) False negative (FN) numbers for the STFT images, waveform images at 40 × 250 pixels and waveform images at 40 × 750 pixels. (**d**) The receiver operating characteristics (ROC) curve of the classification result for the STFT images. (**e**) The ROC curve of the classification result for the waveform images at 40 × 250 pixels. **(f)** The ROC curve of the classification result for the waveform images at 40 × 750 pixels. The area under the curve (AUC) is presented for each ROC curve.

For the 2D image inputs including images of the STFT, raw EEG images with dimensions of 40 × 250 pixels and concatenated raw EEG images with dimensions of 40 × 750 pixels, only the 2D CNNs were applied (Fig. 7a). Compared to classifiers applied to the raw time-series EEG and periodogram results, there were many fewer FPs associated with the 2D image inputs. The FP numbers for the STFT image, 40 × 250 image, and 40 × 750 image were 7,404.0 ± 569.35, 2,576.1 ± 61.64, and 1,814.6 ± 46.23 [F(2, 12) = 83.5, *p* < 0.001, one-way ANOVA], respectively (Fig. 7b). The FN numbers were not different among the input modalities: 30.0 ± 0.94, 31.2 ± 1.11, and 28.2 ± 0.58 for the STFT image, 40 × 250 image, and 40 × 750 image [F(2, 12) = 2.758, *p* = 0.103 by one-way ANOVA], respectively (Fig. 7c). The AUC values were 0.991 (Fig. 7d), 0.993 (Fig. 7e), and 0.998 (Fig. 7f) (*p* = 0.314 for STFT vs. 40 × 250, *p* = 0.003 for the STFT vs. 40 × 750, and *p* = 0.043 for 40 × 250 vs. 40 × 750 by the permutation test). The 40 × 750 images showed significantly better permutation test results compared to all of the other combinations of input modalities and network structures (data not shown). The accuracy, sensitivity, specificity, and F1 score of each classifier were summarized in Table 1.

**Table 1:** Classification results for all the input modalities and network structures.

Input forms	Network structures	Accuracy	Sensitivity	Specificity	F1 score	AUC	FDR
Raw time-series EEG	FCNN	0.985	0.963	0.985	0.031	0.983	0.020
	RNN	0.993	0.966	0.993	0.066	0.989	0.018
	1D CNN	0.996	0.965	0.996	0.118	0.990	0.015
Periodogram	FCNN	0.985	0.963	0.985	0.046	0.984	0.020
	RNN	0.982	0.967	0.982	0.026	0.985	0.024
	1D CNN	0.996	0.962	0.996	0.108	0.989	0.016
Image of STFT	2D CNN	0.998	0.967	0.998	0.194	0.991	0.011
40 × 250 image of EEG	2D CNN	0.999	0.966	0.999	0.407	0.993	0.009
40 × 750 image of EEG	2D CNN	0.999	0.969	0.999	0.492	0.998	0.008
O’shea *et al.*^26^	1D CNN	0.997	0.959	0.997	0.136	0.990	0.012
Zhou *et al*.^25^	1D CNN	0.995	0.957	0.995	0.089	0.989	0.017
Cao *et al*.^29^	2D CNN	0.997	0.962	0.997	0.136	0.990	0.015

AUC: area under the curve, CNN: convolutional neural network, EEG: electroencephalogram, FCNN: fully connected neural network, FDR: false detection rate (n/h), RNN: recurrent neural network, STFT: short-time Fourier transform.

Next, we also tested three recently reported seizure classifiers on our iEEG data (bottom three rows of Table 1). Briefly, O’shea *et al*. built a classifier on the 8-s segments of raw temporal EEG with an 11 layer CNN. Another classifier from Zhou *et al*. was based on 1-s segments of FFT results with a 3 layer CNN. Finally, Cao *et al*. used 2-s segments of the STFT gray image with a 2 layer CNN. AUCs were 0.990, 0.989, and 0.990 for the structures of O’shea *et al*., Zhou *et al*., and Cao *et al*., respectively.

The final purpose of the segment classification is to build a seizure event detector. We generated the seizure events based on the detected seizure segments as described in the methods section. Every classifier missed a same short seizure event, i.e., sensitivity for event detection was 0.997 for all the classifiers. However, there were various numbers of false detections. The FDRs are listed in Table 1. Basically, the FDRs and the numbers of FP segments showed correlation because the seizure events were generated from the results of segment classification. However, since we eliminated much of the discrete FP segments while constructing the seizure events, FDRs were relatively low despite of the many FP segments.

The accuracy, sensitivity, specificity, F1 score, AUC, and FDR for all the classifiers are summarized in Table 1 for comparison. F1 scores were generally very low because of the extreme imbalance in the seizure and non-seizure data, i.e., the FP numbers were much higher than the TP and FN numbers. However, the F1 scores can be greatly improved by the adoption of the 2D raw EEG waveform images.

**Figure 8:**
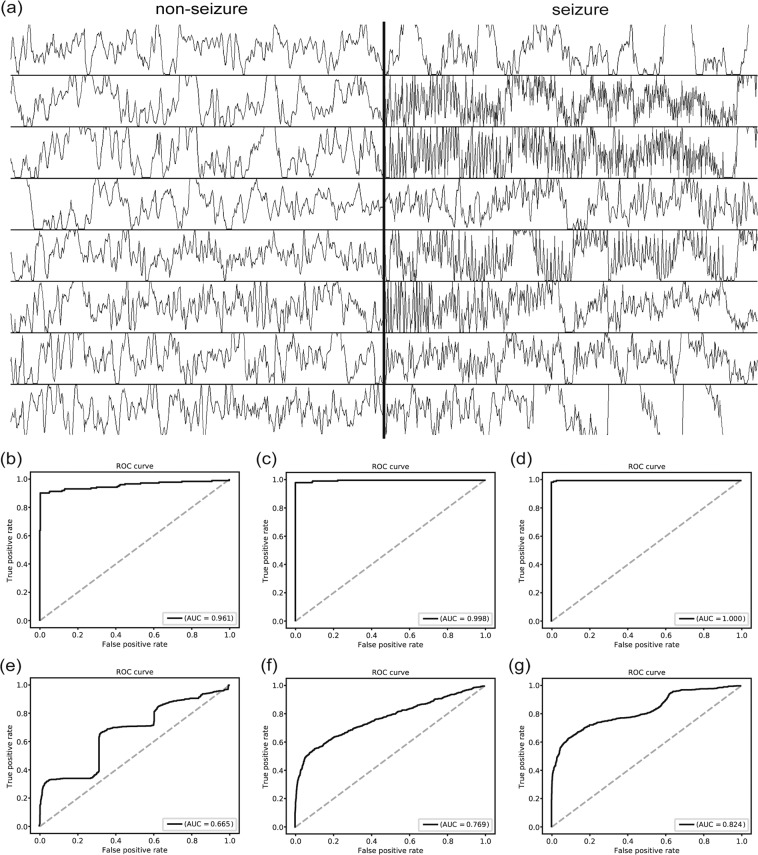
Classification results for the human iEEG. **(a**) Five continuously numbered files were concatenated to form 5-s iEEG segments. Left and right half demonstrate 8 channels of 5-s non-seizure and seizure EEG segments, respectively, of patient 1. The seamless continuation of the EEGs suggested that the files were ordered in a continuous manner. (**b)** The receiver operating characteristics (ROC) curve of the intra-patient classification result for the periodogram results. (**c**) The ROC curve of the intra-patient classification result for the STFT images. (**d**) The ROC curve of the intra-patient classification result for the waveform images at 40 × 250 pixels. **(e**) The ROC curve of the inter-patient classification result for the periodogram results. (**f)** The ROC curve of the inter-patient classification result for the STFT images. (**g**) The ROC curve of the inter-patient classification result for the waveform images at 40 × 250 pixels.

Because we compared the performance of different classifiers with the experimental mice iEEGs, there are a few concerns with respect to our classifiers being applied in the clinical situation. First, these may not generalize to human seizures since we used EEGs obtained from a mouse model of epilepsy. Second, although multi-channel recordings are common in the clinical setting, the mice iEEGs only had a single channel. Thus, we validated our models with a multi-channel human iEEG dataset used for Kaggle seizure detection challenge. The EEGs were supplied as files of 1-s EEG segments with continuous numbering. When we concatenated the five continuous EEG files, they were joined seamlessly (Fig. 8a). Thus, we could build classifiers based on 5-s iEEG segments. The channel number was fixed to 16 because patient 2 and 8 had only 16 channels. Channels 1–16 were used for all the patients other than patient 7, where channels 16–31 were used. AUCs for intra-patient classification were 0.961, 0.998, and 1.000 for the CNN-based classifiers for the periodogram, images of STFT, and images of raw EEG waveforms, respectively (Fig. 8b–d). In case of inter-patient classification, the AUCs were much lower at 0.665, 0.769, and 0.824 (Fig. 8e–g), because the training dataset for human iEEG were much smaller than our mice iEEG dataset. However, the performance order was the same as the mice iEEG, i.e., the performance got better in the order of periodogram, STFT image, and raw EEG waveform image.

## Discussion

Recent studies on deep learning-based seizure detection adopted different input forms, window sizes, network structures, and datasets (Supplementary Table 2). Because of these multiple different factors, it is not possible to directly compare the performance of different approaches for deep learning-based seizure detection. Thus, in the present study, we endeavored to systematically compare how different combinations of input forms and network structures determine the performance of the classifier by using a fixed window size on the same dataset. We included a total of nine different combinations of input modalities and network structures for the comparison.

When the down-sampled raw time-series EEGs were used as the inputs to the FCNN, RNN, and 1D CNN, the CNN resulted in the best AUC with significantly fewer FP results (Fig. 5). The RNN showed an intermediate performance with respect to both the FP numbers and AUC. However, with a periodogram, the RNN yielded overall highest FP results but relatively low FN results compared with the FCNN and CNN (Fig. 6). These results suggest that the information in the periodograms was not discriminative enough for our RNN implementation and resulted in a greater preference for classifying the given data as seizures than non-seizures. Since the periodogram data had only 100 data points, it may not be able to supply enough information to be encoded in the recurrent network, considering the superior performance of an RNN using 500 data points of down-sampled raw time-series EEG. In a recent report, an RNN utilizing 4,096 data points showed good classification results^28^. Thus, RNN implementation for EEG analysis might benefit from a longer sequence of data relative to a shorter sequence. However, the CNN also showed the best performance with a periodogram. These results indicate that a CNN could be the best candidate for the construction of a classifier for 1D input data such as raw time-series EEG, periodogram, and FFT. In a recent paper, Zhou *et al*. compared the performance of a CNN-based classifier, assessing both time domain and frequency domain inputs^25^. In that study, the frequency domain inputs yielded better results with a 1-s window. However, our results showed no difference between the time and frequency domains when a CNN was used. We speculate that the window size may be a critical factor determining the information in the time domain and a 1-s window was too short to contain enough information for the CNN to extract sufficient discriminative features.

Traditionally, CNNs have been widely used to classify 2D images because the CNN is motivated by the neurons in the visual cortex^35,36^. Thus, we tested if a 2D image representation of an EEG could be an adequate input for seizure detection (Fig. 7). When a 2D CNN was applied to the images of an STFT, the FP number was much lower than all of the previous classifiers for the raw EEG time-series and periodogram. Moreover, further improvement was achieved with the raw EEG waveform images as input. We speculate that raw EEG images may reflect the information contained in EEG most faithfully and that the CNN can optimally extract the features of EEG signals from these images. This process clearly resembles the human inspection of EEG from the images on a computer screen. Our classifier for the raw EEG waveform images also showed better results compared to the three recent CNN-based seizure classifiers for the raw temporal EEG^26^, FFT results^25^, and STFT images^29^ (Table 1). More importantly, classifiers for the multi-channel human iEEG also demonstrated a clear performance advantage with the images of the raw EEG waveform (Fig. 8). These results indicated that the current methods were not confined to the animal model of epilepsy but also applicable to the human epilepsy. Thus, we suggest that the image representation of EEG waveforms is the better option as input for CNN-based seizure classifiers. Furthermore, when making a decision on ambiguous EEG seizures, human inspectors may refer to the EEGs before and after the specific seizure event because an ictal EEG shows a different pattern compared to preictal and postictal EEGs^4^. We tried to utilize this strategy by projecting three temporally separated EEGs into one input image. The result was excellent for both the FP number and the AUC. Thus, the concatenation of temporally separated EEGs may provide additional information for distinguishing seizure activity, as expected from the human diagnostic process.

When we used a CNN, the best result was achieved with just two convolutional-pooling layers. In contrast, many researchers implemented more than 6 layers for the CNN^26,37–39^. However, these studies did not include multiple fully connected hidden layers between the convolutional and output layers. In our CNN structures, the two hidden layers may contribute to feature extraction processes and thus diminish the necessity of very deep CNN layers. Otherwise, this may reflect the different requirements based on the receptive field size between different EEG modalities. The receptive fields hierarchically increase with additional convolutional-pooling layers. Because our iEEGs did not require deep CNN architectures, we speculate that the iEEG may contain enough discriminative features even in relatively narrow receptive fields. On the other hand, a scalp EEG may require wider receptive fields because of the averaging effect of the dura and skull^25^. Thus, although it seems clear that the CNN is the best option for seizure detection from iEEGs, detailed structures should be adjusted to obtain the best results depending on the EEG modality.

## Conclusion

To the best of our knowledge, this is the first report investigating how different input modalities and network structures can affect EEG classification results for seizure detection. Our results demonstrated that a CNN can improve the discrimination between seizure and non-seizure EEGs when EEG segments are presented as raw waveform images. Since any kind of extracted features inevitably loose some information present in the original data, more discriminative information might be learned from raw EEG images, provided that the neural network has the potential to extract features contained in the data. Thus, we conclude that the CNN has a remarkably strong potential for the classification of complex signals including EEGs, electrocardiograms, and electromyograms. The CNN works particularly well when an image representation of the signals is provided, although the detailed structure of the CNN should be adjusted depending on signal modality.

## Supplementary information

Supplementary Tables.

## Data Availability

The source codes for the classifiers are available as open-source Python code on GitHub: https://github.com/jajman/CalssifiersforiEEG.
