# Supplementary material for: Comparison of different input modalities and network structures for deep learning-based seizure detection
Source: Sci Rep. 2020 Jan 10;10:122. doi: 10.1038/s41598-019-56958-y (PMC6954227; doi:10.1038/s41598-019-56958-y)
Supplement: Supplementary file 1 — Supplementary Tables. [file 41598_2019_56958_MOESM1_ESM.pdf]

Comparison of different input modalities and network structures for deep learning-based seizure detection

Kyung-Ok Cho[1] · Hyun-Jong Jang[2*]

[1]Department of Pharmacology, Department of Biomedicine & Health Sciences, Catholic Neuroscience Institute, College of Medicine, The Catholic University of Korea, Seoul 06591, South Korea

[2]Department of Physiology, Department of Biomedicine & Health Sciences, Catholic Neuroscience Institute, College of Medicine, The Catholic University of Korea, Seoul 06591, South Korea

*Corresponding author: Hyun-Jong Jang, MD, PhD

Supplementary Table. 1. Network structures applied for the different input modalities.

Input forms		Network structures
Raw time-series EEG	FCNN	Input (500 nodes) – hidden1 (800 nodes) – hidden2 (200 nodes) – output (2 nodes)
	RNN	Input (2 X 250 sequence) – LSTM cell (with 20 units) – averaging of each LSTM output – concatenation (250 nodes) – output (2 nodes)
	1D CNN	Input ([500,1]) – 32 X 5*1 kernel (stride 1) – 2*1 max pooling (stride 2) – 64 X 5*1 kernel – 2*1 max pooling (stride 2) – flatten – hidden1 (512 nodes) – hidden2 (128 nodes) – output (2 nodes)
Periodogram	FCNN	Input (100 nodes) – hidden1 (200 nodes) – hidden2 (50 nodes) – output (2 nodes)
	RNN	Input (2 X 50 sequence) – LSTM cell (with 20 units) – averaging of each LSTM output – concatenation (50 nodes) – output (2 nodes)
	1D CNN	Input ([100,1]) – 32 X 3*1 kernel (stride 1) – 2*1 max pooling (stride 2) – 64 X 3*1 kernel – 2*1 max pooling (stride 2) – flatten – hidden1 (256 nodes) – hidden2 (64 nodes) – output (2 nodes)
Image of STFT	2D CNN	Input ([50,20,1]) – 32 X 3*3 kernel (stride 1*1) – 2*2 max pooling (stride 2*2) – 64 X 3*3 kernel – 2*2 max pooling (stride 2*2) – flatten – hidden1 (256 nodes) – hidden2 (64 nodes) – output (2 nodes)
40 X 250 image of EEG	2D CNN	Input ([40,250,1]) – 32 X 5*5 kernel (stride 1*1) – 2*2 max pooling (stride 2*2) – 64 X 5*5 kernel – 2*2 max pooling (stride 2*2) – flatten – hidden1 (512 nodes) – hidden2 (128 nodes) – output (2 nodes)
40 X 750 image of EEG	2D CNN	Input ([40,750,1]) – 32 X 5*5 kernel (stride 1*1) – 2*2 max pooling (stride 2*2) – 64 X 5*5 kernel – 2*2 max pooling (stride 2*2) – flatten – hidden1 (512 nodes) – hidden2 (128 nodes) – output (2 nodes)

CNN: convolutional neural network, EEG: electroencephalogram, FCNN: fully connected neural network, LSTM: long short-term memory, RNN: recurrent neural network, STFT: short-time Fourier transform.

Supplementary Table. 2. Brief summaries of deep learning-based seizure detection studies

Input forms	Window size	Network structures	Dataset	Reference
Image of FFT result	1 s	Recurrent CNN	CHB-MIT	Thodoroff et al.[38]
Raw temporal EEG	8 s	CNN	CUMH	O'Shea et al.[26]
Image of STFT	2 s	CNN	CHB-MIT	Cao et al.[29]
Raw temporal EEG	90 s	CNN	SCH	Ansari et al.[37]
Raw temporal EEG, FFT	1 s	CNN	CHB-MIT, UHF	Zhou et al.[25]
Raw temporal EEG	23.6 s	CNN	UB	Acharya et al.[39]
Raw temporal EEG	3 s	CNN	UB	Ullah et al.[22]
Image of EEG waveform	5 s	CNN	XMU	Wei et al.[27]
Raw temporal EEG	23.6 s	RNN	UB	Hussein et al.[28]
Raw temporal EEG, periodogram	5 s	FCNN	CUMC	Jang et al.[24]

CHB-MIT: Children's Hospital of Boston-Massachusetts Institute of Technology, CNN: convolutional neural network, CUMC: the Catholic University Medical Center, CUMH: Cork University Maternity Hospital, EEG: electroencephalogram, FCNN: fully connected neural network, FFT: fast Fourier transform, RNN: recurrent neural network, SCH: Sophia Children's Hospital, STFT: short-time Fourier transform, UB: University of Bonn, UHF: University Hospital of Freiburg, XMU: the First Affiliated Hospital of Xinjiang Medical University.
